# Analysis of the Influence of Changing and Fixed Temperatures on the Growth and Pteridine Content in the Head of Adults *Sarcophaga crassipalpis* (Diptera: Sarcophagidae)

**DOI:** 10.3390/ani13152402

**Published:** 2023-07-25

**Authors:** Fernand Jocelin Ngando, Xiangyan Zhang, Hongke Qu, Changquan Zhang, Fengqin Yang, Yakai Feng, Yanjie Shang, Sile Chen, Lipin Ren, Yadong Guo

**Affiliations:** 1 Department of Forensic Science, School of Basic Medical Sciences, Central South University, Changsha 410013, China; 206508005@csu.edu.cn (F.J.N.); zxy196@csu.edu.cn (X.Z.); zcq1105@163.com (C.Z.); may8515@163.com (F.Y.); fyk734@126.com (Y.F.); shangyj@csu.edu.cn (Y.S.); 18216177266@163.com (S.C.); 2 Cancer Research Institute, School of Basic Medical Sciences, Central South University, Changsha 410013, China; quhongke@csu.edu.cn

**Keywords:** *Sarcophaga crassipalpis*, development, variable temperatures, pteridines

## Abstract

**Simple Summary:**

*Sarcophaga crassipalpis* Macquart, 1839 (Diptera: Sarcophagidae) is a flesh fly species of medical, veterinary, and forensic importance. It is often used in the laboratory for several biological studies. In the current research, we investigate its life history under changing temperatures ranging from 15.7 to 31.1 °C, with an average of 24.55 °C, and the relative humidity ranges from 31.4 to 82.8% and at six fixed temperatures of 15, 20, 25, 30, 32, and then 35 °C. Also, pteridine from the head was used to assess adult age grading. Our results revealed that the life history and development rate of *S. crassipalpis* under changing temperatures were very close to those observed at a fixed temperature of 25 °C. The longest and shortest growing times were found at a low rearing temperature of 15 °C and higher temperature of 32 °C, respectively. The pattern of pteridine increase differed depending on the temperature. By employing a changing temperature model in the current study, we aim to provide information on the life history of *S. crassipalpis* that will be useful in future research.

**Abstract:**

Flesh flies (Diptera: Sarcophagidae) are regarded as significant in medical and veterinary entomology, and their development models can be utilized as considerable markers to ascertain the minimum postmortem interval (PMImin). In this research, we explored the growth cycle and larval body length of *Sarcophaga crassipalpis* Macquart 1839 (Diptera: Sarcophagidae) reared under variable temperatures ranging from 15.7 to 31.1 °C, with an average of 24.55 °C and relative humidity ranges from 31.4 to 82.8% and at six fixed temperatures of 15, 20, 25, 30, 32, and then 35 °C. Moreover, pteridine from the head was used to assess adult age grading. Our results allowed us to provide three development models: the isomorphen chart, the isomegalen chart, and the thermal summation models. The time taken for *S. crassipalpis* to complete its development from larviposition to adult emergence at constant temperatures of 15, 20, 25, 30, 32, and 35 °C was 1256.3 ± 124.2, 698.6 ± 15.1, 481.8 ± 35.7, 366.0 ± 13.5, and 295.8 ± 20.5 h, respectively, except 35 °C, where all pupae were unable to attain adulthood. They lasted 485.8 ± 5.4 h under variable temperatures. The minimum developmental limit (*D*_0_) temperature and the thermal summation constant (*K*) of *S. crassipalpis* were 9.31 ± 0.55 °C and 7290.0 ± 388.4 degree hours, respectively. The increase in pteridine content exhibited variations across different temperatures. There was quite a considerable distinction in the pteridine contents of male and female *S. crassipalpis* at 15 °C (*p* = 0.0075) and 25 °C (*p* = 0.0213). At 32 °C and variable temperatures, the pteridine content between female and male *S. crassipalpis* was not statistically divergent. However, temperature and gender remain the main factors influencing the pteridine content in the head of *S. crassipalpis.* We aim to provide detailed developmental data on *S. crassipalpis* that can be used as a valuable resource for future research and PMI estimation.

## 1. Introduction

*Sarcophaga crassipalpis* Macquart, 1839 (Diptera: Sarcophagidae) is a hemisynanthropic *Sarcophagidae* species of medical and veterinary significance. Since 2003, its life cycle has been studied [1]. This species has been known to cause myiasis [2] and has been used in medicolegal entomology in estimating the minimum postmortem interval [3]. It is widely distributed, especially in temperate and tropical regions [4,5]. It is frequently used in the laboratory for a lot of physiological research, including life history, diapause, genetic expression [6,7,8,9,10], and behavior patterns [11], as well as circadian rhythms [12,13,14]. Temperature governs entire aspects of ectothermic organisms, especially their growth and development [15]. Since insects live in thermally variable environments, they are subjected to various thermal regimes, including extremely high and low temperatures, which can impact their physiology and life story [15,16,17,18]. Nonetheless, the mechanisms regulating the development of *S. crassipalpis* under varying temperature conditions remain poorly understood. Little research has been performed on the life history of this species, including its development under non-diapause conditions [19,20], constant temperatures [21], reproductive time [22,23] and fecundity [24], eclosion rhythm [8], and pupal diapause [8,25,26], as well as the effects of food and weather conditions on its growth processes [10]. The findings show developmental plasticity in *S. crassipalpis* with different development patterns in populations from different geographical locations in the USA, China, and Turkey.

Most of the species belonging to the *Sarcophagidae* family exhibit a slow growth rate at low temperatures and a fast growth rate at high temperatures, as evidenced by the development patterns exhibited by *Sarcophaga dux* Thomson, 1869 (Diptera: Sarcophagidae) [27], *Sarcophaga peregrina* (Robineau-Desvoidy, 1830) (Diptera: Sarcophagidae) [28], *Sarcophaga (Liopygia) argyrostoma* Robineau-Desvoidy, 1830 [29], *Sarcophaga ruficornis* (Fabricius, 1794) [30,31], and *Wohlfahrtia nubia* Wiedemann, 1830 (Diptera: Sarcophagidae) [32]. *Sarcophaga africa* had a slow life cycle, with a 1.66-day difference between the rainy and winter seasons and a rapid one during the summer [33]. Shang et al. found that, under fluctuating temperatures (18–36 °C and 22–30 °C), *S. peregrina* had a longer development and a lower pupariation rate and emergence rate than those observed at constant temperatures [34]. 

Some discrepancy was observed in the growth rates of insects maintained under controlled laboratory conditions with a constant temperature and those subjected to variable temperatures in their natural habitat. For instance, it has been reported that *Protophormia terraenovae*, Robineau-Desvoidy, 1830 (Diptera: Calliphoridae) developed quicker at a higher changing temperature and slower at the lowest temperature range [35]. Niederegger et al. recorded, under daily low and optimum fluctuating temperatures (5–29 °C), the faster development of *S. argyrostoma* (Diptera: Sarcophagidae) and *Lucilia illustris* (Meigen, 1926) (Diptera: Calliphoridae) and lower development of *Calliphora vicina* (Robineau-Desvoidy) and *Calliphora vomitoria* (Linnaeus, 1758) (Diptera: Calliphoridae) [36], whereas Chen and collaborators reported that *Aldrichina grahami* (Aldrich, 1930) (Diptera: Calliphoridae) had a cold thermal preference with a low thermal threshold of 3.41 ± 0.48 °C [37]. Byrd and Butler reported that, under fluctuating temperatures, *Sarcophaga haemorrhoidalis* (Fallen) (Diptera: Sarcophagidae) had a maximal thermal preference of 30 °C [38]. Brent and Spurgeon [39] found non-significant developmental durations of *Lygus Hesperus* (Hemiptera: Miridae) observed at moderate and high fluctuating temperatures of 22 °C and 29 °C, respectively. Mironidis [40] observed that the longevity and fecundity of adult *Helicoverpa armigera* (Lepidoptera: Noctuidae) were decreased at variable temperatures ranging from 17.5 to 32.5 °C. One of the reasons for these various effects is that fluctuating temperatures may delay the development process at all stages, with survival and longevity gradually decreasing as temperatures decrease and increase [41]. Moreover, significant deviations from the standard temperature range may adversely impact the thermophysiological capabilities of insects [41,42]. It is vital to recognize that the frequency of extreme weather events is projected to rise in the future, with potentially significant consequences for insect populations [43]. Consequently, more research is required to elucidate the developmental processes of insects under conditions of temperature variability. 

Pteridines are heterocyclic substances derived from the pyrimidine-pyrazine and are 2-amino-4-hydroxy derivatives that accumulated in the eyes of adult flies over time [44]. They are commonly known as ocular pigments and serve as a filter for UV protection [44,45] and as a pathway of nitrite elimination [44,46]. In the other words, these are biochemical metabolics that can be quantified and utilized for calculating the ages of insects [47,48]. Temperature is a factor that affects pteridine levels. It promotes an increase in the amount of pteridine as adult flies age [44,49]. To date, the quantitative pteridine levels of forensically important dipteran species, including *C. vicina* (Robineau-Desvoidy) (Diptera: Calliphoridae) [45], *Musca domestica* Linnaeus, (Diptera: Muscidae) [50], *Boettcherisca peregrina* (Robineau-Desvoidy) [51], *Lucilia sericata* (Meigen) (Diptera: Calliphoridae) [44], and *Chrysomya megacephala* (Fabricius) (Diptera: Calliphoridae) [52], have been investigated. 

In this research, the baseline developmental data for *S. crassipalpis* were obtained at six fixed and variable temperatures. In addition, the pteridine content in the head of *S. crassipalpis* was quantified and analyzed at the same variable temperature conditions and three constant temperatures (15, 25, and 32 °C) to assess adult age grading. The findings have significant implications for utilizing this species in estimating the minimum postmortem intervals during forensic examinations.

## 2. Materials and Methods

### 2.1. Settlement of Laboratory Specimen and SAMPLING

Wild adults of *S. crassipalpis* were captured on pork bait using Nylon nets for trapping flies in Xi Hu Park in Changsha City (28°12′ N; 112°58′ E), Hunan Province, China, in September and October 2021. Before raising, they were anesthetized at −20 °C for 1–2 min and identified under Zeiss AxioCam 208 color microscopy using morphological keys by a medicolegal entomologist expert [53] and confirmed using molecular techniques by performing a polymerase chain reaction (PCR) of the long cytochrome oxidase subunit I (COI) gene [54]. The species were reared according to the fly culture methodology previously described by Zhang et al. [55]. Adult *S. crassipalpis* were cultivated in a rearing nylon box and placed in an artificial climate cage (250A GPL, Shen Zhen Ren Gong. Ltd., Tianjin, China). The weather conditions were 25.0 °C temperature, 70% relative humidity, and 12:12 h of light/dark photoperiod cycles. The flies were fed a diet consisting of water and milk powder and were reared to the fifth generation before starting the experiments. Before the commencement of the study, the total number of adult *S. crassipalpis* was maintained at 1000–2000 specimens. 

**Figure 1:**
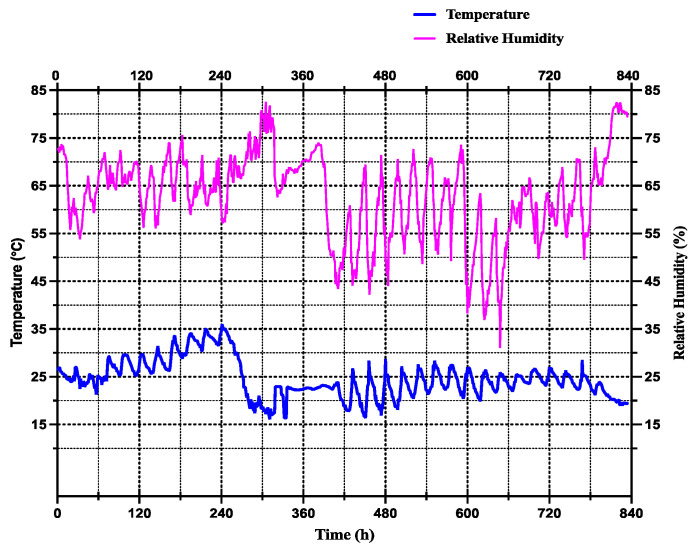
Variable temperatures and relative humidity were recorded based on the meteorologic conditions occurring in Hunan Province, China, using a GPS temperature and moisture data logger (GPS-6, Elitech Co., Ltd., Jiangsu, China) in the months of September and October 2022. The blue line represented the average daily fluctuating temperatures, while the purple one symbolized the average daily fluctuating relative humidity.

Six artificial climate incubators (LRH-250-GSI, Taihong Co., Ltd., Shaoguan, China) were set at fixed temperatures of 15 °C, 20 °C 25 °C, 30 °C, 32 °C, and then 35 °C, with 70% RH and a photoperiod 12:12 h L/D cycle, while the flies did not undergo eclosion at a temperature of 35 °C. As in the variable temperature conditions the rearing occurs outside, the variable temperatures were determined based on meteorological conditions in Hunan Province, Southeast China. A GPS temperature and humidity data logger (GPS-6, Elitech Co., Ltd., Jiangsu, China) was used to record the minimum, average, and maximum ambient temperatures of 15.7 °C, 24.55 °C, and 31.1 °C, respectively, during September and October 2022. The relative minimum and maximum humidity ranged from 31.4% to 82.8% (Figure 1).

### 2.2. Evaluation of Lifespan and Measurement of Larval Body Length

Approximately 50 g of pig lungs were put into Petri dishes and introduced into a fly-rearing box to stimulate larviposition. After 2 h, approximately 2000–3000 larvae deposited by gravid female *S. crassipalpis* were collected. Each batch containing 400–500 larvae was reared in a plastic bowl with a relative quantity of pig lungs [56]. The bowls were then introduced into fly-breeding cages covered with 2 cm of wet sand until pupation [56]. Larvae rearing and sampling were conducted under both variable (outside) and constant temperatures, as previously described.

Larvae were monitored every 8 h from the first instar to wandering and every 12 h from pupation to adult eclosion. Fresh pieces of pork lungs were provided 1 to 3 times per day based on consumption [56]. Eight or ten larvae were collected every 8 h until wandering and then treated in hot water at 90 °C for thirty seconds, as previously described [57]. The larvae were conserved in a centrifuge tube containing 75% ethanol and stored at −20 °C [58]. The larval instar was established by examining the number of splits in the posterior spiracle using Zeiss AxioCam 208 color microscopy [59]. The body lengths were measured using an electronic Vernier caliper (Meinaite, Shanghai, China). The different stages of development, including wandering, pupation, and eclosion, were noted during the experiment [56]. Each experiment was repeated with five biological replicates. A total of 4090 larvae were sampled: 1250 in 15 °C, 680 in 20 °C, 600 in 25 °C, 450 in 30 °C, 320 in 32 °C, 350 in 35°C, and 440 in the variable temperature group.

### 2.3. Pteridine Extraction

Adult *S. crassipalpis* were collected at three constant (15, 25, and 32 °C) and variable (15.7 °C to 31.1 °C) temperatures when approximately 50% of the pupae emerged into adults and were designated as zero days. Eight adults (four males and four females) were then sampled every 48 h for fourteen days. The samples were preserved in 2 mL centrifuge tubes at −80 °C for the subsequent analysis. A global number of 32 adult males and 32 adult females were collected per group. The experiment was repeated with three biological replicates.

Pteridine extraction and fluorescence analysis were performed using the modified methods previously described [44,49,51,60,61,62]. The flies were taken out from the −80 °C refrigerator and unfrozen, and the sex of each individual was noted. Their heads were decapitated, detached from their bodies, and transferred into 2 mL centrifuge tubes. The mouthpieces were carefully removed from each head using dissecting tweezers (Code No. TST-11; R’DEER tools, Hong Kong Robust Deer Tools Co. Limited, Hong Kong) under an AxioCam 208 color microscope (Carl Zeiss Microscopy GmbH, Jena, Germany). Each head capsule was weighed utilizing precision scales (Code No. TP-100D; Xiangyi Balance Equipment, Changsha, China) and returned to the 2 mL centrifuge tubes. The head capsules were mixed in 600 μL of 1 M Tris-HCl buffer at pH 8.0 [45]. A maceration bead was added to each 2 mL microcentrifuge tube containing a head capsule and macerated using a Tissuelyser-24 (Ling Xin Industrial Development Co. Ltd., Shanghai, China) at 50.00 hertz for 35 s. The tubes were then centrifuged in a Microfuge® 20R Centrifuge (Ref. B31614; Beckman Coulter Euro Center SA, Nylon 1, Nyon, Switzerland) and centrifugated at 6000× *g* for 5 min in 4 °C; then, 150 μL of the supernatant was loaded into a 96-well cell culture plate (Code No. 11510; LABSELECT®, Beijing Labgic Technology Co., Ltd., Beijing, China) and covered with aluminium foil. The fluorescence intensity was gauged instantly after stimulation. The pteridine fluorescence was gauged in an EnSpire® Multimode Plate Reader (PerkinElmer, Waltham, MA, USA) at a transmitter frequency of 482 nanometers with the agitation set at 360–450 nanometers. The capacity of the released light was expressed in the relative fluorescence units (RFU) [45,52].

### 2.4. Statistical Analysis

GraphPad Prism 9.4.1 and IBM SPSS Statistics 26 were used to perform the data analysis; One-way ANOVA examined the consequence of the temperature on the total duration of the life story [37]. Nonlinear regression was utilized for analyzing the correlation between the larval body length and feeding period [63,64]. To define the equation for the PMImin estimation, the larval body length was used as the independent variable while the time after larviposition was the dependent variable and inversely [56]. The reviewed regression pattern outlined by Ikemoto and Takai [65], as reported by Mukesh et al. [66]: D = K + Tm, where D represents development time, K the thermal constant, and Tm the low thermal threshold, was utilized for the analysis of the correlation between the developmental rate and accumulation degree hours (ADH) at each developmental stage and the total developmental times [27]. The mathematical equations were obtained using GraphPad Prism version 9.4.1, Boston, MA, USA. The slope and y-intercept of the linear regression equation for each stage were both utilized to define the developmental threshold temperature *D*_0_ and thermal accumulated constant K, respectively [67]. 

The age of the adults of *S. crassipalpis* was defined based on the methods prior described [45,49]. The adult age was expressed as the time after eclosion in accumulated degree days (ADD) (ADD = temperature in Celsius degrees × age in days) [68] without subtraction of the minimum temperature threshold [45]. Using IBM SPSS Statistics version 26, a log10 transformation of pteridine fluorescence and ADD was utilized to perform a linear regression and write the regression equations for females and males in each temperature group [45]. Paired *t*-tests and two-way ANOVA were utilized to analyze differences in the pteridine levels between different temperatures and sexes.

## 3. Results

### 3.1. Development Time at Fixed and Changing Temperatures

The developmental cycle of *S. crassipalpis* was observed under variable temperatures ranging from 15.7 to 31.1 °C (mean: 24.55 °C) (Figure 1) and at 6 fixed temperatures (15, 20, 25, 30, 32, and 35 °C). The results indicate that the length of the developmental cycle reduced with the rising temperature from the early immature stage to pupae (Table 1).

A one-way ANOVA analysis at a *p*-value inferior to 0.05 demonstrated a considerable distinction between the means of total developmental durations for constant vs. variable temperatures (*F* = 200.2, df = 28, *p* < 0.0001, *R*^2^ = 0.9775). However, comparisons between each group of constants vs. variable temperatures (VT) showed varying results. According to Tukey’s multiple comparison test and one-way ANOVA at alpha = 0.05, there was no statistical significance between the total developmental duration at 25 °C vs. VT (df = 23, *p* = 0.9999). In contrast, significant differences were found between total developmental durations at 15 °C and VT (df = 23, *p* < 0.0001), 20 °C and VT (df = 23, *p* <0.0001), 30 °C and VT (df = 23, *p* = 0.0377), and 32 °C vs. variable temperature (df = 23, *p* = 0.0005) (Supplementary Figure S1).

### 3.2. Lifespan and Isomorphen Diagrams

However, at 35 °C, while larvae completed the active feeding, wandering, and pupariation stages, they did not emerge as adult flies. This indicates that 35 °C is a higher lethal thermal for *S. crassipalpis* (Table 1). Additionally, the life cycle duration was reduced from 1256.3 h at 15 °C to 295.8 h at 32 °C and was recorded as 485.8 h at VT (Table 1). According to Tukey’s multiple comparison test and one-way ANOVA at alpha = 0.05, there is a considerable variation between the development times at 25 °C and 15 °C (df = 23, *p* < 0.0001), at 25 °C and 20 °C (df = 23, *p* < 0.0001), at 25 °C and 30 °C (df = 23, *p* = 0.0314), and at 25 °C and 32 °C (df = 23, *p* = 0.0003) (Supplementary Figure S2).

**Figure 2:**
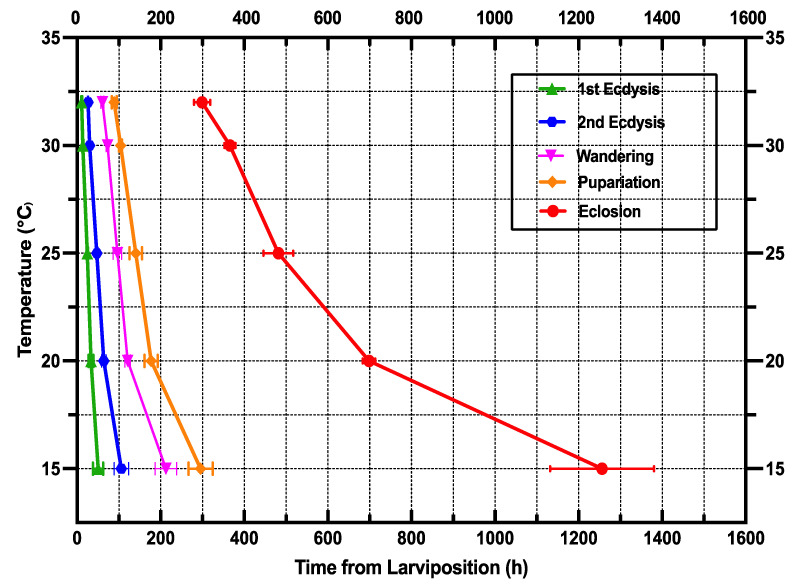
Isomorphen diagram of *S. crassipalpis*. The development time of each stage (first ecdysis, second ecdysis, wandering, pupation, and eclosion) was charted with the time from larviposition to the onset of each development time. Each curve represents a developmental event, and the error bar corresponds to the standard deviation of each event.

The isomorphen diagram was designed based on the time of all developmental phases from larviposition to adult emergence (x-axis) vs. different fixed temperatures (y-axis), where each line characterizes larval body changes, and the distances between lines describe the developmental stages [69]. An increase in temperature from 15 to 32 °C caused the time between each growth stage (first ecdysis, second ecdysis, wandering, pupation, and eclosion) to shrink and the distances between lines to decrease (Figure 2).

### 3.3. Thermal Accumulated Models

**Figure 3:**
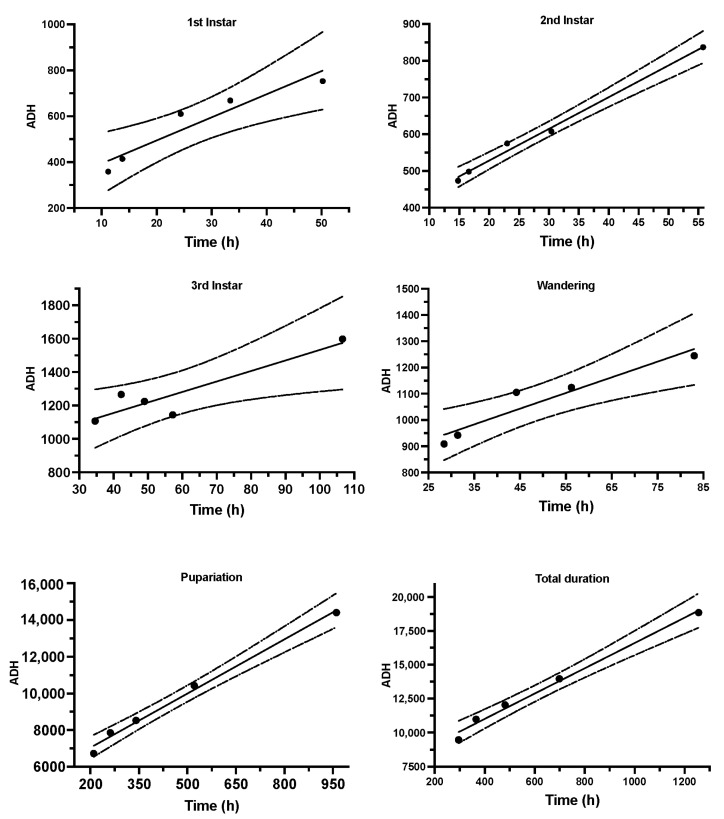
Thermal accumulated models for five developmental phases and total developmental duration of *S. crassipalpis*. The strong line corresponds to the regression line, and the dashed line corresponds to a 95% confidence interval.

According to the correlation between the developmental times of *S. crassipalpis* from larviposition to adult emergence (x-axis) and accumulated degree hours (y-axis), six thermal summation patterns were plotted using linear regression examination (Figure 3). The coefficient of determination (*R*^2^) of all thermal accumulated models was ≥0.90, demonstrating that all data matched relatively similarly to the linear models (Table 2). Utilizing the revised regression model suggested by Ikemoto and Takai [65], the developmental threshold temperature (*D*_0_) and thermal summation constants (*K*) were estimated to be 9.31 ± 0.55 °C and 7290.0 ± 388.4 degree hours, respectively. In addition, the thermal requirements (*K*) were 293.0 ± 58.1, 355.5 ± 13.9, 904.2 ± 100.7, 774.4 ± 56.8, and 5066.0 ± 272.4 degree hours at the first, second, and third immature stages, as well as wandering and pupal stages, respectively (Table 2). The development of larvae to adult emergence was slowest at 15 °C and quickest at 32 °C, as is usual in other sarcophagids.

### 3.4. Variations in Larval Body Length Measurement and Isomegalen Graphs

**Figure 4:**
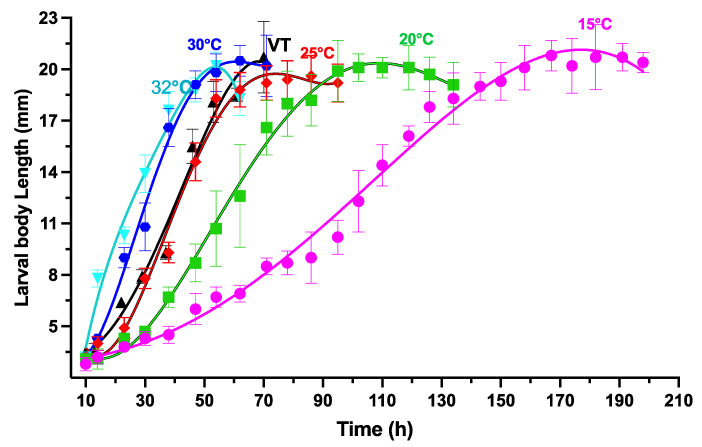
Variations in larval body length of *S. crassipalpis* over time (h) after larviposition at diverse fixed temperatures. The vertical bars symbolize the standard deviation.

Figure 4 displays the data of larval body length changes over time after larviposition in different constant and variable temperatures. Larvae grew faster at increasing temperatures (15–32 °C). The disparity in growth rates between 15 °C and 25 °C was significant, but it reduced as the temperatures reached 30 °C to 32 °C. The fourth-order polynomials models at each temperature group were used to define the equations characterizing the larval body length changes over time after larviposition. The larval body length was used as the independent variable, while the time after larviposition was the dependent variable. At 15 °C, 20 °C, 25 °C, 30 °C, 32 °C, and then VT, the average optimum larval body length was 20.8, 20.1, 20.2, 20.1, 20.2, and 20.7 mm, respectively.

The equations in Table 3 exhibit the fluctuations in larval body length (*L*) with the elapse of time (*T*), with larval body length as the dependent variable and time after larviposition as the independent variable. The coefficient of determination (*R*^2^), F value, and *p*-value suggest that the models have a good correlation with the data. The equations in Table 4 display the connection between time (*T*) and larval body length (*L*) by using time after larviposition as the dependent factor and the larval body length as the independent factor.

**Figure 5:**
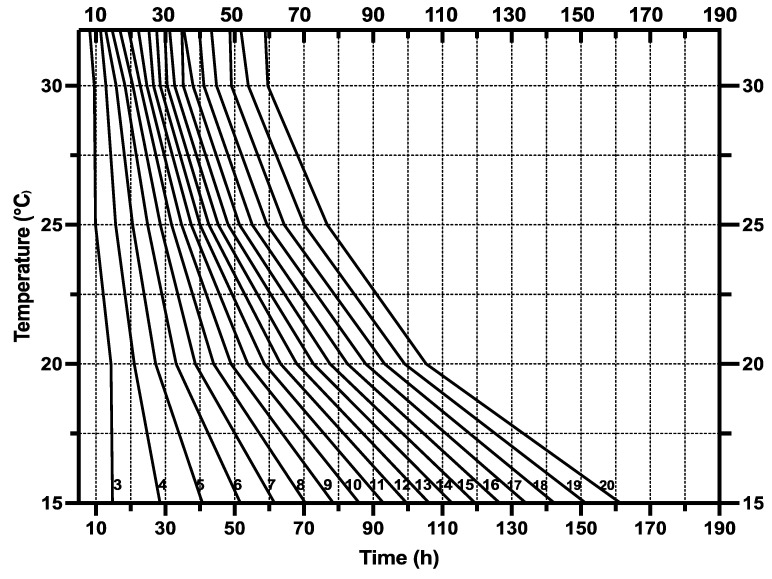
Isomegalen graph of *S. crassipalpis* larvae from larviposition to peak feeding phase. Time (h) was charted against temperature, and each line corresponds to the developmental larval length of 3–20 mm, with size indicated by the number at the lower left of each contour.

The Isomegalen diagram (Figure 5) was constructed by plotting the time elapsed from larviposition to the peak feeding stage on the x-axis and z-axis, while the constant temperatures were on the y-axis. The lower boundary of each contour displays a range of larval body lengths from 3 mm to 20 mm.

### 3.5. Effect of Temperature Variation on the Content of Pteridine

**Figure 6:**
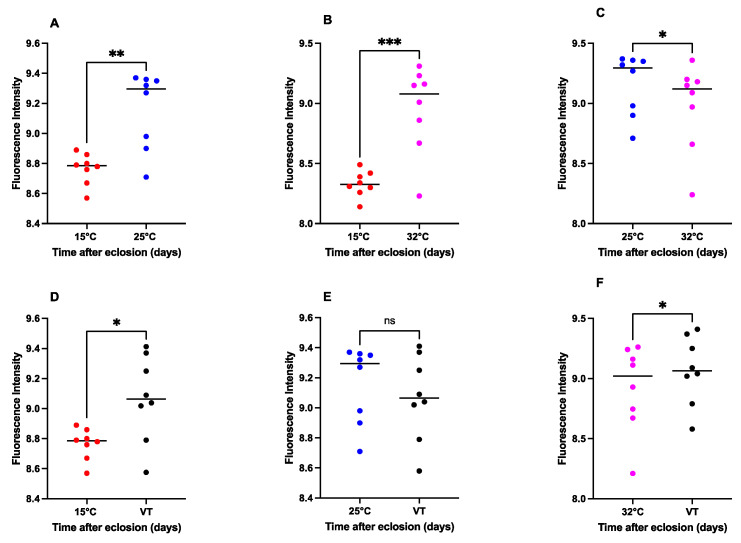
Comparison of the pteridine content of *S. crassipalpis* with different temperatures ((**A**): 15 °C vs. 25 °C; (**B**): 15 °C vs. 32 °C; (**C**): 25 °C vs. 32 °C; (**D**): 15 °C vs. VT; (**E**): 25 °C vs. VT, and (**F**): 32 °C vs. VT). The number of asterisks indicates the significant difference between different temperature groups: ***: *p* < 0.001; **: *p* < 0.01; *: *p* < 0.05; ns: not significant.

The pteridine concentrations exhibited statistically significant differences in 15 °C against 25 °C (*p* = 0.0019, t = 4.847, df = 7), 15 °C against 32 °C (t = 6.375, df = 7, *p* = 0.0004), 15 °C against VT (t = 3.419, df = 7, *p* = 0.0112), 25 °C against 32 °C (t = 3.412, df = 7, *p* = 0.0113), and 32 °C against VT (t = 2.667, df = 7, *p* = 0.0321). However, there was no considerable distinction between 25 °C and VT (t = 2.065, df = 7, *p* = 0.0778) (Figure 6).

**Figure 7:**
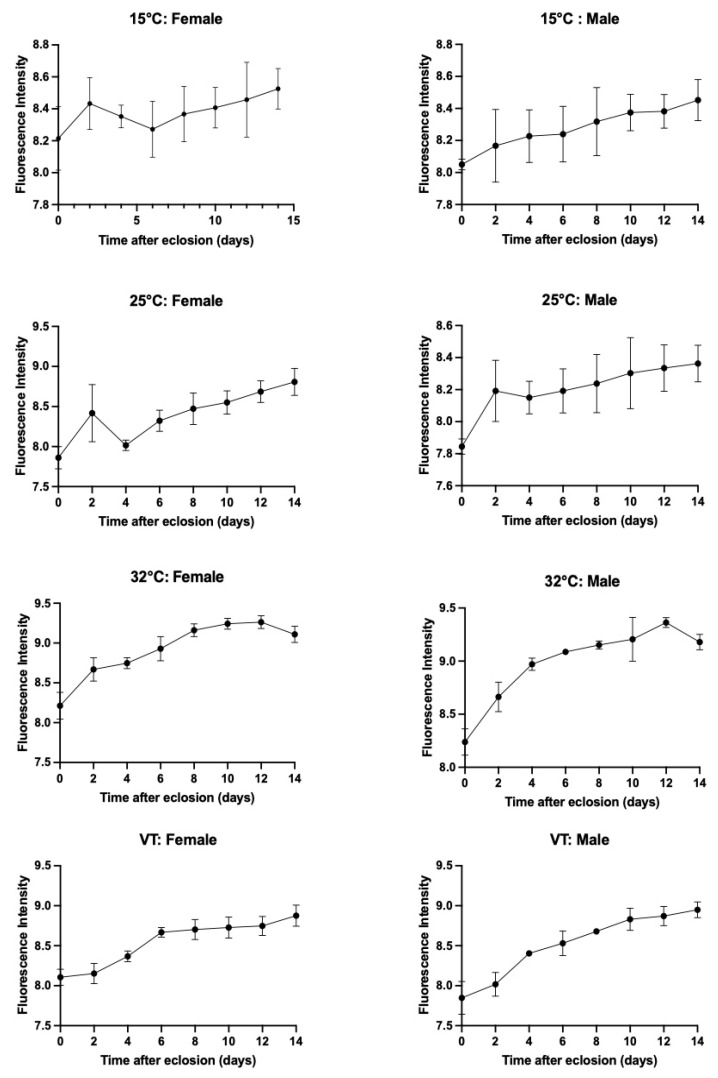
Accumulated pteridines in the heads of females and males of *S. crassipalpis* when raised to three different constant and variable temperatures. The pteridine fluorescence intensity (RFU) for each sex (y-axis) was plotted against time after eclosion (x-axis) in days.

An increase in the pteridine content was observed over time following eclosion, as illustrated in Figure 7. Nevertheless, the pattern of this increase varied across different temperatures.

**Figure 8:**
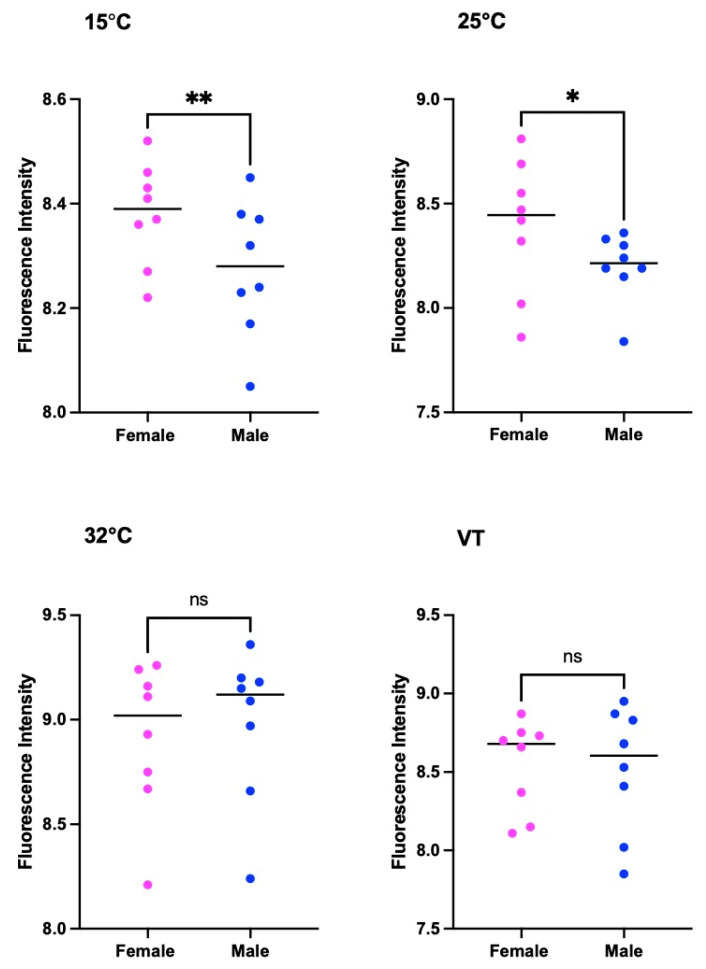
Comparison of the pteridine content of *S. crassipalpis* between females and males under different temperatures. The number of asterisks indicates the significant difference between different temperature groups. **: *p* < 0.01; *: *p* < 0.05; ns: not significant.

There was a substantial difference in the pteridine concentrations between females and males of *S. crassipaplis* at 15 °C (t = 3.719, df = 7, *p* = 0.0075) and 25 °C (t = 2.952, df = 7, *p* = 0.0213). Nevertheless, there was no statistical variation in the pteridine level between males and females at 32 °C (*p* = 0.0827, t = 2.023, df = 7) and under variable temperatures (*p* = 0.619, t = 0.5205, df = 7) (Figure 8).

In addition, the scrutiny of the pteridine content in the heads of adult male and female *S crassipalpis* exhibited a linear regression that correlated with the rising log10 of ADD and, therefore, with time after eclosion (Figure S4). The accumulated pteridine concentration could be used to predict the age of adult *S. crassipalpis*. Across all experiment conditions, the head capsule weight did not have a statistically reliable effect; the *p*-value was over 0.05. Except for females collected at 15 °C (*R*^2^ = 0.503), the index of determination of all regression patterns was *R*^2^ ≥ 0.700, indicating that all data fit relatively well to the linear models (Table 5). Thus, the age of male and female adults of *S. crassipalpis* can be determined by applying the equations from Table 5.

## 4. Discussion

This study provides the examination of developmental times for the flesh fly species *S. crassipalpis*, which holds medical and veterinary significance, particularly in forensic medicine. The investigation was conducted under variable and constant laboratory temperatures in Changsha City, Hunan Province, China. We provided developmental data, including isomegalen and isomorphen diagrams, thermal summation models, and a pteridine content analysis of adult heads, to facilitate the use of this species in estimating PMImin.

The investigational results showed that the variable temperature group generated a moderated life cycle duration and a moderated rate in comparison with the control group of a fixed temperature. Under variable temperatures, the total developmental duration of *S. crassipalpis* was 485.8 ± 5.4 h, relatively similar to the developmental duration observed under a constant temperature of 25 °C (469.8 ± 27.1 h). However, the development was slower under constant temperatures of 15 °C (1256.3 ± 124.2 h) and 20 °C (698.6 ± 15.1) than under variable temperature conditions (485.8 ± 5.4 h), while development was relatively long under variable temperatures compared with the developmental duration under 30 °C (366.0 ± 13.5 h) and 32 °C (295.8 ± 20.5), with significant differences. These data indicated that the mean temperature of variable temperatures was nearer to the optimal constant temperature. In a comparison to the effect of variable temperatures vs. constant temperatures on *S. crassipalpis,* it appeared that, under the low constant temperature groups, *S. crassipalpis* grew more slowly, moderately under the optimum temperature and greater under high temperatures.

Since there is not yet a published study on the consequence of variable temperatures on the lifespan of *S. crassipalpis*, the present research findings were discussed with the available literature on other taxa. In the current study, we found that variable temperatures can influence the development rates and durations of *S. crassipalpis*. Analogously, Dadour et al. [70] found that *Hydrotaea rostrata* (Diptera: Muscidae) had slower developmental rates under winter conditions than under a summer regime. Clarkson et al. [71] reported that *P. terraenovae* (Diptera: Calliphoridae) developed more quickly in the early immature stages under fixed temperatures than under fluctuating temperatures.

Similarly, an investigation on the development of larvae of different forensic flies was conducted by Niederegger et al. [36] under naturally changing (5–29 °C) and invariable (13 °C) temperatures, with invariable temperatures designed as the average of changing temperatures. They discovered that, at variable temperatures, *S. argyrostoma* and *L. illustris* developed more rapidly than *C. vicina* and *C. vomitoria*. In 2013, Warren and Anderson [35] conducted other research on the influence of unstable temperatures on the life history of *P. terraenovae* and found that its growth was accelerating at 4–28 °C higher fluctuating temperatures, reasonable at 9–23 °C changing temperatures, and slowest at 16 °C stable temperatures. Interestingly, this discrepancy in development rate is thought to have been caused by the rate summation effect, as temperature variations above the mean tend to raise the rate relatively more than they can lower it. Recently, Chen et al. [37] investigated the life history parameters of *A. grahami* (Diptera: Calliphoridae) under stable and changing temperatures. They found that *A. grahami* developed slowly under changing temperatures ranging from 6 to 20 °C, according to the natural meteorological conditions, than under stable temperatures ranging from 8 to 36 °C. These correlated with our findings in the present study. Sert et al. [72] analyzed the impact of fixed and changing temperatures on the post-feeding time of *S. argyrostoma* (Diptera: Sarcophagidae) and found a similar intrapuparial development with a non-significant difference between adult emergence times at fixed temperatures (25 °C) and variable temperature conditions. Although this result concerned exclusively the intrapuparial stage, it was similar to the observation made on the growth time of *S. crassipalpis* under variable temperatures vs. 25 °C in the current research.

A thorough understanding of insect development parameters under fluctuating temperatures can aid in addressing various challenges, including accelerated, delayed, or unchanged developmental rates [37]. The results of experiments on the impact of variable temperatures on insect development can be related to the climatic conditions under which the models were chosen and the thermophysiological capacity of each species to adapt to fluctuating temperatures. Even though data from studies on the development of insects under different constant temperatures have been successfully used to estimate PMI, several studies found it inconceivable to use this model to investigate the developmental parameters of insects that naturally live under different variable temperature conditions [42,73,74]. Thus, ignoring variable temperatures in real investigations can result in significantly incorrect estimates of the PMI [36]. Furthermore, variable temperatures should be factored into growth prediction models [37].

Several studies on various diapause parameters in insects have been conducted on *S. crassipalpis* [6,26,75,76,77]. However, very few studies have examined its development time [10,19]. In our study, *S. crassipalpis* developed successfully at 15 to 32 °C and ceased to accomplish its growth at 35 °C, similar to the thermal conditions chosen by Bulut et al. [10] to analyze the impacts of tissue type and invariable temperatures on the life story of *S. crassipalpis* in Turkey. In contrast, the developmental rate of Turkey’s colony was lower at 32 °C for specimens fed bovine minced meat, bovine tongue, and chicken heart, and not all pupae reached adult emergence when fed minced meat [10]. This is different from the current study colony, which developed successfully at 32 °C while being reared on pig lung tissue. This disparity could be attributed to the rearing tissues [10].

The growth time of the immature stages of *S. crassipalpis* in the present study at 15 °C is shorter than those fed with bovine tongues and bovine minced meat in Turkey [10]. However, those reared on chicken heart tissue at 15 °C developed 1.81 days faster than the Chinese colony (our study). At 20 °C, the larvae of our colony developed faster than those of the Turkish colony [10]. Interestingly, when comparing the duration larval stage at 25 °C and 30 °C, the results from the present study are very similar to the Turkish study [10], except when the larvae were reared on chicken heart tissue. In contrast, at 32 °C, the growth period of the maggots in China, the present study was significantly shorter than in Turkey [10]. The successful development of *S. crassipalpis* in low temperature conditions can be attributed to its cold tolerance capacity. Chen et al. [78] found that a short acclimation at 0 °C allowed its larvae, pupae, and adults to subsist at −10 °C by increasing their hemolymph osmolality and glycerol levels, as well as the membrane fatty acids, necessary for low temperature tolerance [79].

In the current investigation, the developmental period of the pupal phase decreased with the growing temperatures, similar to the study carried out by Bulut et al. [10]. However, the pupa’s development times were slower than in Turkey, except for 30 °C, which was similar to the Turkish specimen fed with chicken heart tissue [10]. At 32 °C, the present investigation found that the period of the pupal phase was shorter than in Turkey. The developmental events (from larvae to pupae) for *S. crassipalpis* varied among the temperatures [19]. These disparities reflect the plasticity of developmental rates across populations, as well as the different food and feeding techniques, which may influence the development time of insects [56].

In our study, the higher constant temperatures we exposed *S. crassipalpis* to were 30, 32, and 35 °C. Although the larvae accomplished their development at 30 °C and 32 °C with total growing durations of 366 h and 295.8 h, respectively, unfortunately, all pupae ceased to reach the adult phase at 35 °C. This suggests that the longer exposure time at this temperature resulted in insufficient development of muscle contraction motor patterns for successful adult emergence [80]. Similarly, Bulut et al. [10] found that *S. crassipalpis* can complete their developmental time at 30 °C, with lower average developmental times to achieve the developmental cycle than at 32 °C and at 35 °C, where all larvae failed to reach the pupal phase. These findings suggested that 32 °C could be close to the upper growing threshold temperature for *S. crassipalpis*. These observations supported those made by Nassu et al. [31] on the development time of *Microcerella halli* (Engel 1913) (Diptera: Sarcophagidae) raised under diverse constant temperatures. They found that larvae of *M. halli* raised at 35 °C attained the pupal phase much later than other groups but never reached the adult stage. Contrary, Joseph et al. [24] discovered that an exposition for one or two hours at the suboptimal (−10 °C) and supraoptimal (40 °C and 45 °C) temperatures decreased the fecundity in both male and female *S. crassipalpis* but did not affect the larvae and pupae. 

Our study found that temperature significantly impacted the development period of *S. crassipalpis* from the immature phases to adult emergence. These findings supported those of Bulut et al., who discovered that temperature and tissue type had a significant impact on the immature stage and pupal survival, as well as the adult weight of *S. crassipalpis* [10]. Also, Chen et al. [19] noted that the span from leaving the larval nutritional source to the beginning of pharate adult development showed the highest response to temperature variations, showing that physiological processes occurring at this time are particularly susceptible to temperature control [81]. Research has indicated that laboratory equipment and conditions, moisture, and photoperiods may have an impact on the development process, but the temperature is the predominant factor that determines the growth and development of insects [27]. Temperature plays a significant role in phenotypical variations, which may lead to geographical variations. Furthermore, the temperature favors adaptation to the geographical area. Therefore, it is important to prioritize assessing the aforementioned factors, recording data precisely, and designing the experiment cautiously to form a reliable benchmark for the period of fly development [82].

The pteridine content in the heads of *S. crassipalpis* is a reliable technique for determining the age of adult necrophagous species for the PMI estimation. In the current study, there is a logical correlation between the time after eclosion, thus the age of the adult *S. crassipalpis*, and the pteridine intensity, as well as between an increased pteridine concentration and different temperatures. Previous studies have proven a positive interaction between the pteridine content and the age of necrophagous flies, including *Stomoxy calcitrant* [83,84], *Glossina morsitrans* [61], *Cochliomyia hominivorax* [60], *Chrysomya bezziana* [85], *Lucilia sericata* [44,86], *Aldrichina grahami* [87], *Musca domestica* [50], *Chrysomya megacephala* [52], *Cochliomyia macellaria, Phormia regina* [49], *Boettcherisca peregrina* [51], and *Calliphora vicina* [45].

Our findings indicate that temperature is a crucial factor in the increase of pteridine. The pattern of this increase varied across different temperatures, suggesting that the metabolic activity of pteridine increased at higher temperatures. This is coherent with the results of Zhu et al. [52], who found that the content of pteridine was twice as high in the high-temperature group as opposed to the low-temperature group. This affirmative thermal impact on the pteridine concentration is consistent in *B. peregrina, Stomoxys calcitrans,* and *M. domestica* as well [50,51,84]. Contrary to our results, Bernhardt et al. [45] found a non-significant relationship between temperature and the rise of the pteridine levels in the head of *C. vicina.* We additionally observed that the head capsule had no impact on the pteridine levels, which is in agreement with the findings of Bernhardt et al. [45], who found that the head weight did not influence the pteridine fluorescence. 

Our research found a substantial linear correlation between the pteridine content and age of male and female adults of *S. crassipalpis* at different constant and variable temperatures. This corresponds to the conclusion of Zhu et al. [51], who observed a significant linear interaction between pteridine fluorescence and age in male and female adults of *B. peregrina* (Diptera: Sarcophagidae) raised in different constant temperatures. In our study, there was a considerable variation in the content of pteridine between female and male *S. crassipalpis* at 15 °C and 25 °C. Nevertheless, we found no statistical distinction in the pteridine content between female and male *S. crassipalpis* at 32 °C and variable temperatures. This contrasts with previous research, which showed that male flies exhibited higher pteridine levels than female flies (*L. sericata*, *M. domestica*, and *Calliphora erythrocephala*) [50,88]. We suspect that the lack of gender difference in the morphology of *S. crassipalpis* eyes and increasing temperature may be crucial factors in our findings (Figure S3 and Figure 8). The low *R*^2^ values observed in females sampled at 15 °C may be related to the decreased metabolic activity of the specimens raised at this temperature. Similarly, Cammack et al. [49] also discovered low indexes of determination in both male and female *Phormia regina* reared at 5.40 °C and suggested that this finding can be linked to reduced metabolic activity, which is the result of poor physical activity, and the reduced consumption of food and water of this species. Because pteridines are a byproduct of purine metabolism, a decrease in metabolic activity would culminate in a decrease in the pteridine concentration levels [49]. For a more precise estimation of the PMI, it is suggested that the pteridine content should be used in combination with other aging methods [45].

Our results allowed us to generate the following development patterns, including isomorphen diagram (Figure 2), isomegalen diagram (Figure 5), and the thermal accumulated model (Figure 3), commonly used by forensic entomologists to determine the minimum period after death. An isomorphen graph provides data on age ranges based on the times of the developmental stages (x-axis) against the temperatures (y-axis) to estimate the PMImin [37]. Hence, the oldest individual’s age found on the corpse and the average ambient temperature close to the experimental temperatures are essential for using this method to estimate the PMImin [89,90]. Nevertheless, the simplicity of this method may compromise the precision of the results [37,91]. Some researchers discovered that an isomegalen diagram is more accurate to estimate the PMImin than an isomorphen diagram [91,92], because it is the only method that sets out changes in the larval body length between the larviposition and peak feeding [64]; unfortunately, it does not apply when the larvae shrink naturally [37,91]. The method to kill and preserve larvae collected should be taken into account when using the larval body length. In this study, larvae were killed in boiled water at 90° for 30 s to cause the larvae to expand to their full extent and preserved in 75% ethanol, as recommended by Adams and Martin [57]. However, the larva should be measured immediately after killing in boiling water before preservation [57,93]. Therefore, some authors have suggested that the accumulated degree hours (ADH) technique is preferable to the two earlier methods, given its applicability in natural settings with varying temperatures [89,94]. Nonetheless, the weakness of this method is related to the large variations between the higher and lower developmental temperature limits [37,91]. In addition, Wu et al. [73] found that, when making quantitative predictions, it is not possible to use ADH models, since the connection between temperature and the rate of development is not totally correlative.

All of these models could be used to estimate the PMImin; however, the biology of the insects and the climatic parameters under which they are dynamic should be considered [37]. In addition, to provide an accurate PMI, Shang et al. [28] suggested the use of a multimethod combination, as well as a combination of multidisciplinary specialists, to analyze and interpret data from forensic investigations [95]. Therefore, further research is still required to test other methods using this species.

## 5. Conclusions

This research offers the initial experimental laboratory information on the lifespan of *S. crassipalpis* at variable and constant temperatures in China for the minimum postmortem interval estimation (PMImin). Our findings showed that the life history and development rate of *S. crassipalpis* under variable temperatures were very close to those observed at a fixed temperature of 25 °C. The longest and shortest growing times were found at a low rearing temperature of 15 °C and higher temperature of 32 °C, respectively. However, all pupae failed to emerge at 35 °C, suggesting that the upper developmental threshold temperature for *S. crassipalpis* may be close to this temperature. The pattern of pteridine increase varied across different temperatures. In addition, most of the total developmental durations exhibited significant differences between variable and constant temperatures. By utilizing a fluctuating temperature model in our study, we aimed to provide detailed developmental data on *S. crassipalpis* that can serve as a valuable resource for future research.

## Figures and Tables

**Table 1:** Mean (SD ±) development time (h) of *S. crassipalpis* at six fixed and variable temperatures (VT).

Development Stages (°C)	First Instar	Second Instar	Third Instar	Wandering	Pupa	Total Duration
15	50.2 ± 12.3	55.8 ± 7.2	106.6 ± 10.2	83.0 ± 11.2	960.7 ± 100.1	1256.3 ± 124.2
20	33.4 ± 5.7	30.4 ± 4.8	57.2 ± 6.3	56.2 ± 11.3	521.4 ± 22.8	698.6 ± 15.1
25	24.4 ± 1.7	23.0 ± 1.7	49.0 ± 8.2	44.2 ± 14.6	341.2 ± 42.0	481.8 ± 35.7
30	13.8 ± 1.3	16.6 ± 1.3	42.2 ± 3.3	31.4 ± 0.5	262.0 ± 10.1	366.0 ± 13.5
32	11.2 ± 1.3	14.8 ± 0.4	34.6 ± 3.0	28.4 ± 4.0	210.2 ± 20.6	295.8 ± 20.5
VT	12.0 ± 2.7	19.8 ± 4.9	48.5 ± 11.6	49.8 ± 6.7	355.8 ± 13.6	485.8 ± 5.4
35	9.0 ± 1.1	14.6 ± 1.8	38.4 ± 8.8	33.4 ± 8.9 *	-	-

* All pupae failed to reach adult stage.

**Table 2:** Mean (±SE) of the developmental threshold temperature (*D*_0_) and thermal summation constants (*K*) for five developmental stages and the total developmental period of *S. crassipalpis* and the coefficient of determination (*R*^2^) of the thermal summation models.

Developmental Stages	*K* (Degree Hours)		*D*_0_ (°C)		
	Mean	SE	Mean	SE	*R* ^2^
First instar	293.0	58.1	10.06	1.93	0.90
Second instar	355.5	13.9	8.64	0.44	0.99
Third instar	904.2	100.7	6.29	1.59	0.84
Wandering	774.4	56.8	5.97	1.08	0.91
Pupa	5066.0	272.4	9.86	0.51	0.99
Total duration	7290.0	388.4	9.31	0.55	0.99

**Table 3:** Equations, *F* values, *p*-values, and coefficient of determination (*R*^2^) of the relationship between the body length (*L*, mm) of *S. crassipalpis* larvae and the time after larviposition (*T*, h) at constant and variable temperatures.

Temperature (°C)	Equation	*F*	*p*	*R^2^*
15	*L* = −7.789 × 10^−6^ × *T*3 + 0.002*T*2 − 0.62*T* + 3.851	2651.8	<0.0001	0.970
20	*L* = −2.823 × 10^−5^ × *T*3 + 0.005*T*2 − 0.021*T* + 2.436	908.8	<0.0001	0.954
25	*L* = −6.216 × 10^−5^ × *T*3 + 0.007*T*2 − 0.056*T* + 1.567	891.5	<0.0001	0.958
30	*L* = −9.507 × 10^−5^ × *T*3 + 0.006*T*2 + 0.322*T* − 0.322	1081.0	<0.0001	0.974
32	*L* = −9.368 × 10^−5^ × *T*3 + 0.005*T*2 − 0.359*T* − 0.842	796.1	<0.0001	0.975
VT	*L* = −9.311 × 10^−6^ × *T*3 − 0.003*T*2 + 0.596*T* − 2.855	2777.1	<0.0001	0.990

**Table 4:** Equations, *F* values, *p*-values, and coefficient of determination (*R*^2^) of the relationship between the time after larviposition (*T*, h) and the body length (*L*, mm) of *S. crassipalpis* larvae at constant and variable temperatures.

Temperature (°C)	Equation	*F*	*p*	*R^2^*
15	*T* = 0.027*L*3 − 1.046*L*2 + 20.022*L* − 36.621	1402.0	<0.0001	0.945
20	*T* = 0.009*L*3 − 0.326*L*2 + 8.628*L* − 8.854	284.4	<0.0001	0.866
25	*T* = 0.019*L*3 − 0.628*L*2 + 9.469*L* − 13.423	337.1	<0.0001	0.897
30	*T* = 0.013*L*3 − 0.379*L*2 + 5.569*L* − 4.178	328.1	<0.0001	0.920
32	*T* = −0.007*L*3 + 0.302*L*2 − 0.805*L* + 10.113	133.7	<0.0001	0.870
VT	*T* = 0.016*L*3 − 0.377*L*2 + 4.818*L* − 1.568	260.5	<0.0001	0.903

**Table 5:** Regression analysis of the pteridine fluorescence intensity (RFU) against times (ADD) for female and male *S. crassipalpis* at three constant (15 °C, 25 °C, and 32 °C) and variable (mean = 24.55 °C) temperatures. Times in accumulated degree per day (ADD) were used as the dependent variable and pteridines fluorescence intensity as the independent variable. F = female, M = male, y = predicted age hours, and x = pteridines fluorescence intensity.

Temperatures	Sex	Regression Equations	*R* ^2^	*p*	*F*
15 °C	F	y = 6.51x (±2.64) − 21.765 (±9.62)	0.503	<0.049	6.082
	M	y = 6.72x (±0.61) − 22.236 (±2.22)	0.952	<0.0001	118.203
25 °C	F	y = 2.33x (±0.62) − 6.338 (±2.27)	0.700	<0.01	14.014
	M	y = 5.16x (±0.708) − 16.228 (±2.52)	0.899	<0.0001	53.248
32 °C	F	y = 2.44x (±0.255) − 7.177 (±0.989)	0.939	<0.0001	91.861
	M	y = 2.43x (±0.211) − 7.200 (±0.823)	0.957	<0.0001	133.265
VT	F	y = 2.90x (±0.42) − 8.585 (±1.587)	0.885	<0.0001	46.034
	M	y = 2.19x (±0.214) − 5.926 (±0.792)	0.946	<0.0001	104.956

## Data Availability

The data and code presented in this study are contained in the manuscript and available on request from the corresponding author; therefore, they are not filed in a public repository.

## Supplementary Materials

The following supporting information can be downloaded at: https://www.mdpi.com/article/10.3390/ani13152402/s1: Figure S1. Tukey’s multiple comparison test, one-way ANOVA at alpha = 0.05 of the total developmental durations between constant vs. variable temperatures. The significant differences among developmental durations are indicated with asterisks. Figure S2. Tukey’s multiple comparison test, one-way ANOVA at alpha = 0.05 of the total developmental durations between the constant temperature control group (25 °C) vs. other temperature groups. The significant differences among the developmental durations are indicated with asterisks. Figure S3. Sex differences in the morphology of *S. crassipalpis* eyes: (A) female and (B) male. Figure S4. Log10 of time (ADD) after eclosion plotted against log10 of the mean pteridine fluorescence (RFU) for males and females at different temperatures ((A) 15 °C, (B) 25 °C, (C) 32 °C, and (D) mean variable temperatures (24.55 °C).
