# Supplementary figures and images for: Analysis of the Influence of Changing and Fixed Temperatures on the Growth and Pteridine Content in the Head of Adults Sarcophaga crassipalpis (Diptera: Sarcophagidae)
Source: Animals (Basel). 2023 Jul 25;13(15):2402. doi: 10.3390/ani13152402 (PMC10417853; doi:10.3390/ani13152402)

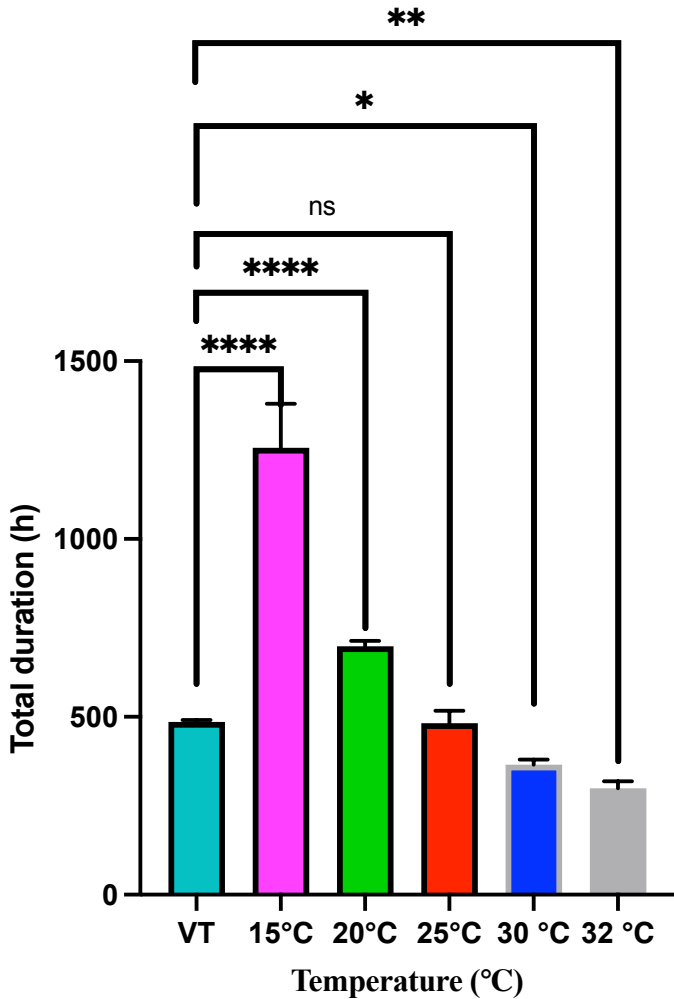

Supplement: Supplementary file 1 [file animals-13-02402-s001.zip › Figure S1.pdf]

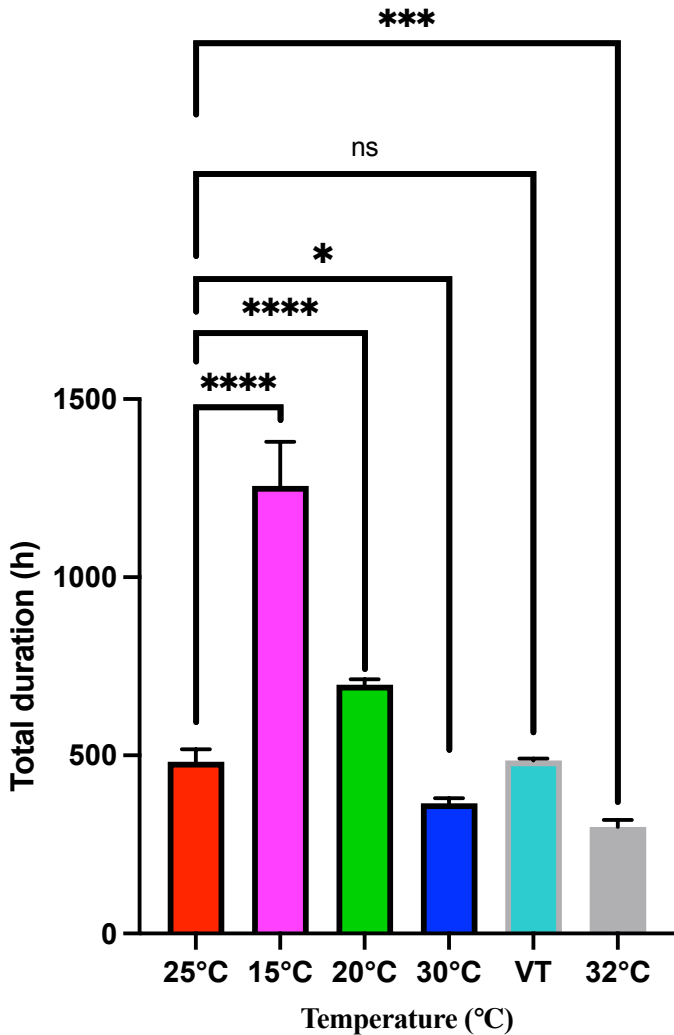

Supplement: Supplementary file 1 [file animals-13-02402-s001.zip › Figure S2.pdf]

A

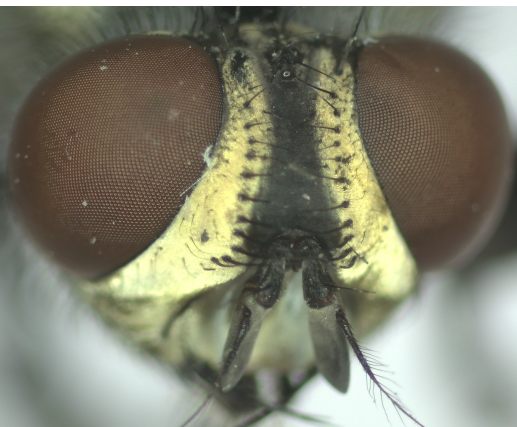

B

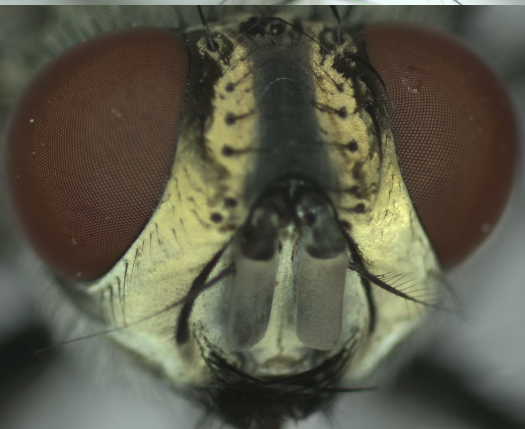

Supplement: Supplementary file 1 [file animals-13-02402-s001.zip › Figure S3.pdf]

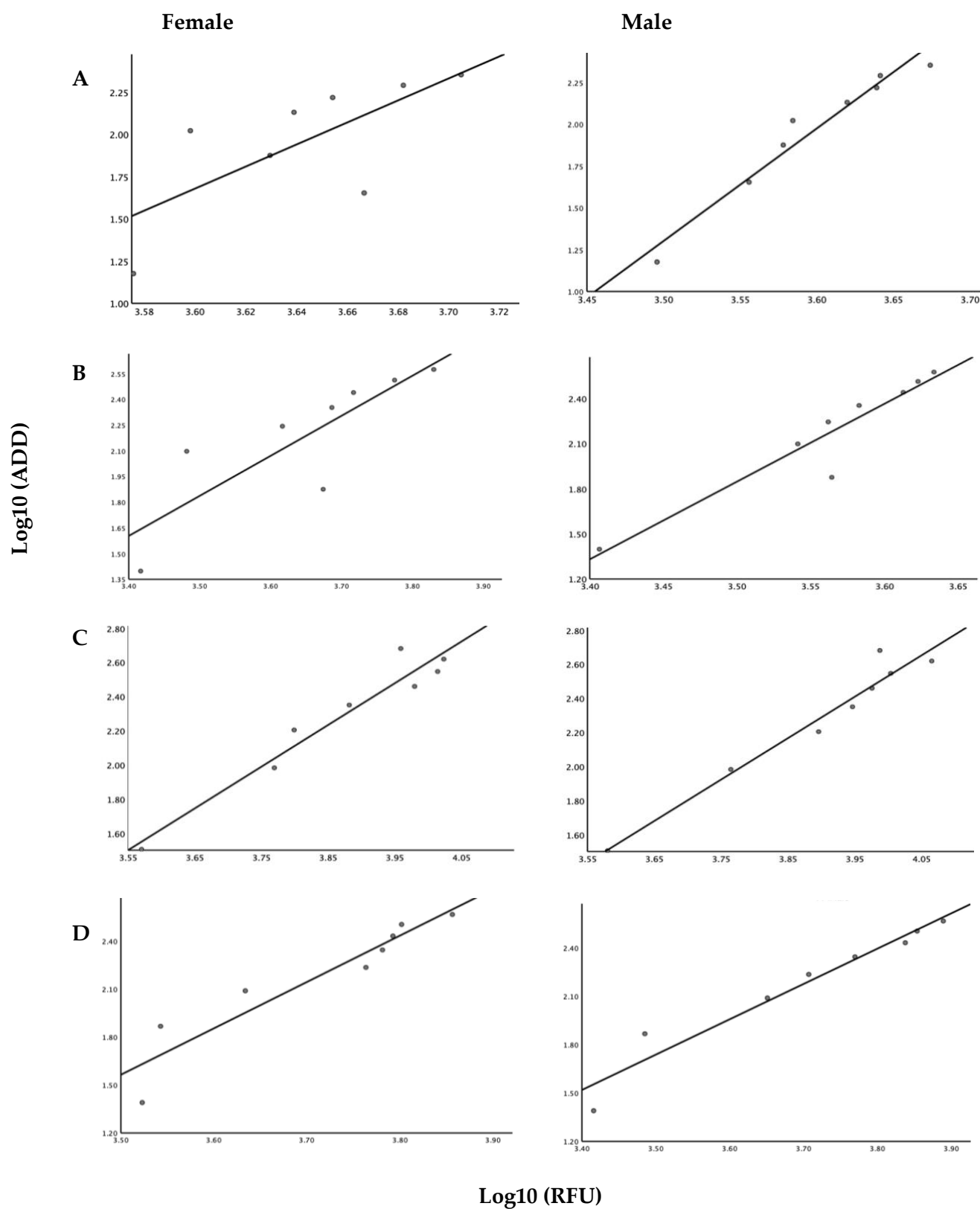

Supplement: Supplementary file 1 [file animals-13-02402-s001.zip › Figure S4.pdf]
